# Prevalence, clinical features, and outcomes of SARS-CoV-2 infection in pregnant women with or without mild/moderate symptoms: Results from universal screening in a tertiary care center in Mexico City, Mexico

**DOI:** 10.1371/journal.pone.0249584

**Published:** 2021-04-22

**Authors:** J. Arturo Cardona-Pérez, Isabel Villegas-Mota, A. Cecilia Helguera-Repetto, Sandra Acevedo-Gallegos, Mario Rodríguez-Bosch, Mónica Aguinaga-Ríos, Irma Coronado-Zarco, Moisés León-Juárez, Diana Aguilar-Ayala, María Yolotzin Valdespino-Vázquez, Elsa Romelia Moreno-Verduzco, María Antonieta Rivera, Carolina Valencia-Contreras, María de Lourdes Gómez-Sousa, Mario Solis-Paredes, Brenda Frías-Madrid, César Velasco-Téllez, Juan Carlos Rodriguez-Aldama, Valeria Avila-Sosa, Rafael Galván-Contreras, Ricardo Figueroa-Damian, Manuel Cortés-Bonilla, Guadalupe Estrada-Gutierrez, Salvador Espino-y-Sosa, Claudine Irles

**Affiliations:** 1 Dirección General, Instituto Nacional de Perinatología Isidro Espinosa de los Reyes, Mexico City, Mexico; 2 Unidad de Enfermedades Infecciosas y Epidemiología, Instituto Nacional de Perinatología Isidro Espinosa de los Reyes, Mexico City, Mexico; 3 Departamento de Immunobioquímica, Instituto Nacional de Perinatología Isidro Espinosa de los Reyes, Mexico City, Mexico; 4 Departamento de Medicina Materno-Fetal, Instituto Nacional de Perinatología Isidro Espinosa de los Reyes, Mexico City, Mexico; 5 Subdirección de Ginecología y Obstetricia, Instituto Nacional de Perinatología Isidro Espinosa de los Reyes, Mexico City, Mexico; 6 Departamento de Genética y Genómica Humana, Instituto Nacional de Perinatología Isidro Espinosa de los Reyes, Mexico City, Mexico; 7 Subdirección de Neonatología, Instituto Nacional de Perinatología Isidro Espinosa de los Reyes, Mexico City, Mexico; 8 Departamento de Anatomía Patológica, Instituto Nacional de Perinatología Isidro Espinosa de los Reyes, Mexico City, Mexico; 9 Subdirección de Servicios auxiliares de Diagnóstico, Instituto Nacional de Perinatología Isidro Espinosa de los Reyes, Mexico City, Mexico; 10 Unidad de Cuidados Intensivos del Recién Nacido, Instituto Nacional de Perinatología Isidro Espinosa de los Reyes, Mexico City, Mexico; 11 Unidad de Cuidados Inmediatos al Recién Nacido, Instituto Nacional de Perinatología Isidro Espinosa de los Reyes, Mexico City, Mexico; 12 Departamento de Posgrado e Investigación, Instituto Nacional de Perinatología Isidro Espinosa de los Reyes, Mexico City, Mexico; 13 Departamento de Salud Mental, Instituto Nacional de Perinatología Isidro Espinosa de los Reyes, Mexico City, Mexico; 14 Departamento de Fisiología y Desarrollo Celular, Instituto Nacional de Perinatología Isidro Espinosa de los Reyes, Mexico City, Mexico; 15 Departamento de Infectología e Inmunología, Instituto Nacional de Perinatología Isidro Espinosa de los Reyes, Mexico City, Mexico; 16 Dirección Médica, Instituto Nacional de Perinatología Isidro Espinosa de los Reyes, Mexico City, Mexico; 17 Dirección de Investigación, Instituto Nacional de Perinatología Isidro Espinosa de los Reyes, Mexico City, Mexico; 18 Subdirección de Investigación Clínica, Instituto Nacional de Perinatología Isidro Espinosa de los Reyes, Mexico City, Mexico; Indiana University School of Medicine, UNITED STATES

## Abstract

The perinatal consequences of SARS-CoV-2 infection are still largely unknown. This study aimed to describe the features and outcomes of pregnant women with or without SARS-CoV-2 infection after the universal screening was established in a large tertiary care center admitting only obstetric related conditions without severe COVID-19 in Mexico City. This retrospective case-control study integrates data between April 22 and May 25, 2020, during active community transmission in Mexico, with one of the highest COVID-19 test positivity percentages worldwide. Only pregnant women and neonates with a SARS-CoV-2 result by quantitative RT-PCR were included in this study. Among 240 pregnant women, the prevalence of COVID-19 was 29% (95% CI, 24% to 35%); 86% of the patients were asymptomatic (95% CI, 76%-92%), nine women presented mild symptoms, and one patient moderate disease. No pregnancy baseline features or risk factors associated with severity of infection, including maternal age > 35 years, Body Mass Index >30 kg/m^2^, and pre-existing diseases, differed between positive and negative women. The median gestational age at admission for both groups was 38 weeks. All women were discharged at home without complications, and no maternal death was reported. The proportion of preeclampsia was higher in positive women than negative women (18%, 95% CI, 10%-29% vs. 9%, 95% CI, 5%-14%, *P*<0.05). No differences were found for other perinatal outcomes. SARS-CoV-2 test result was positive for nine infants of positive mothers detected within 24h of birth. An increased number of infected neonates were admitted to the NICU, compared to negative neonates (44% vs. 22%, *P*<0.05) and had a longer length of hospitalization (2 [2–18] days vs. 2 [2–3] days, *P*<0.001); these are potential proxies for illness severity. This report highlights the importance of COVID-19 detection at delivery in pregnant women living in high transmission areas.

## Introduction

Coronavirus Disease 2019 (COVID-19), caused by the novel severe acute respiratory syndrome coronavirus 2 (SARS-CoV-2), has now reached more than 87,736,000 confirmed cases and 1,892,256 deaths. As of April 2020, Mexico has the fourth-highest number of COVID-19 associated deaths worldwide (https://coronavirus.jhu.edu/map), and at the time of this study also one of the highest positivity rates; more than 40% at the national level and 32.7% for Mexico City. National epidemiological reports have shown an elevated number of maternal deaths (2.3% to 15%) among women with COVID-19 infection [1] (https://www.gob.mx/salud/documentos/informes-semanales-para-la-vigilancia-epidemiologica-de-muertes-maternas-2020, Mexican Ministry of Health). Pregnant women represent a unique population, which to date still have unanswered questions regarding COVID-19 infection and its perinatal consequences [2–6]. Recently, available data supports that pregnant women are at increased risk for severe illness from COVID-19 compared to non-pregnant women, as updated by the CDC on December 23, 2020 (https://www.cdc.gov/coronavirus/2019-ncov/need-extra-precautions/people-with-medical-conditions.html#pregnancy). Furthermore, several studies have shown that COVID-19 during pregnancy is associated with adverse outcomes, such as preterm birth and stillbirth [7–9], reviewed by [10]. Joining the Global effort to elucidate the impact of COVID-19 during pregnancy, and as previously implemented by several countries [11,12], as of April 19^th^, 2020, universal screening was established for all pregnant women attending the Instituto Nacional de Perinatología Isidro Espinosa de los Reyes, a large referral Level 3 care center for Perinatology in Mexico City, with more than 4000 deliveries per year. To date, this center is the only facility to implement universal screening for COVID-19 in pregnant women nationally. It is important to mention that public hospitals in Mexico were classified as COVID-19 or non-COVID-19 according to their capacity to admit or not patients with severe COVID-19 symptoms or illness. As this health care center was classified as non-COVID-19, only obstetric related conditions without severe COVID-19 symptoms were admitted at the hospital for delivery. Therefore, this report intends to provide information regarding the epidemiological/clinical characteristics and outcomes of pregnant women admitted for obstetric reasons who tested positive or negative for SARS-CoV-2 as part of asymptomatic screening practices at delivery (excluding severe symptomatic patients) during the pandemic peak in one of the most affected cities worldwide.

## Material and methods

### Study design and patients

The Ethics in Research Committee (ERC) of the National Institute of Perinatology (Instituto Nacional de Perinatología Isidro Espinosa de los Reyes, INPer) approved this study, (#2020-1-31) conducted according to the principles of the Declaration of Helsinki, and followed the Strengthening of the Reporting of Observational Studies in Epidemiology (STROBE) guidelines (www.equator-network.org). The ERC approved that no consent informed form was needed for this study since the data were analyzed anonymously from electronic medical records. This work is part of a larger institutional study untitled: “Epidemiological and clinical characterization of SARS-CoV-2 infection during the perinatal period”, led by Dr. J. A. Cardona-Pérez (# 2020-1-32). The universal screening was implemented on April 19, 2020, to all obstetric admissions at the INPer, a large tertiary level non-COVID-19 center in Mexico City; patients with severe COVID-19 symptoms were referred to COVID-19 hospitals. Therefore, this analysis was limited to mainly asymptomatic pregnant women and some women with mild/moderate symptoms.

Maternal admissions were stratified into two groups based on delivery: scheduled C-section or obstetric emergency during labor (such as abortion, premature rupture of membranes, preeclampsia, preterm birth, among others). All women went to COVID triage upon arrival, where a nasopharyngeal swab was obtained, and the following data were recorded: temperature, cardiac frequency, blood pressure, pulse oximetry, and COVID-19-compatible symptoms. Asymptomatic and symptomatic women were classified based on the absence of symptoms or the presence of at least one specific symptom consistent with COVID-19. Women with severe symptoms (dyspnea and SpO_2_<93%, and tachypnea with a respiratory rate ≥ of 30 breaths per minute, or suspected severe pneumonia) according to [13,14] and WHO classification (https://www.who.int/publications/i/item/report-of-the-who-china-joint-mission-on-coronavirus-disease-2019-(covid-19))) were referred to a COVID-19 hospital. SARS-CoV-2 positive mothers and positive neonates were isolated in COVID-19 restricted areas but were not roomed together. Non-infected neonates were admitted to a non-COVID-19 section. Mothers positive for COVID-19 used N-95 mask during labor, C-section, or anytime they interacted with their newborns.

These series of control-cases were retrospectively collected between April 22, 2020, and May 25, 2020.

#### Data collection tool and database

Data on SARS-CoV-2 positive and negative cases were electronically reported by a multidisciplinary team. The research team used google spreadsheets to collect the data. The database was developed based on the information needs of each of the participating hospital areas and validated for analysis. The database generates automatic reports that allowed the analysis of epidemiological behavior at the hospital level. The collaborative environment enables the different clinical areas to enter the information with real-time access to it by the researchers simultaneously. Data included: epidemiological, anthropometric, clinical, obstetric, management, delivery methods, outcomes from maternal-fetal, and neonatal patients (from electronic medical records). All patients’ data were deidentified and analyzed anonymously.

### Molecular diagnosis for SARS-CoV-2 infection

Nasopharyngeal swabs were taken from all pregnant women as part of universal screening practices at the delivery hospitalization (with mild/moderate or without COVID-19 symptoms). Reverse Transcriptase- quantitative Polymerase Reaction (RT-qPCR) test for SARS-CoV-2 was performed at the Department of Immunobiochemistry.

The test was accredited by the referral National Institute for Diagnosis and Epidemiological Reference (Instituto Nacional de Referencia Epidemiológica Dr. Manuel Martínez Baez, Mexico City, Mexico). The SARS-CoV-2 RNA was detected following the instruction of La Charité, Berlin protocol [15].

At birth, neonates were not universally screened, and the test was performed in some infants of positive mothers or some negative mothers who presented symptoms, such as fever or headache.

### Eligibility criteria

Two groups of women were admitted for delivery: scheduled C-section or labor (obstetric emergency). Of these, only those with a molecular test for SARS-CoV-2 were included in this study. Newborns with a SARS-CoV-2 PCR result within 24h of birth and a COVID-19 test result for their mother were also included in this work.

### Case-control definitions and classification of maternal infection during pregnancy

Pregnant women were classified into cases (SARS-CoV-2 RT-qPCR positive result) or controls (negative RT-qPCR result). Classification of SARS-CoV-2 infection during pregnancy was the following (according to [16]): symptomatic or asymptomatic mother with confirmed detection of the virus by PCR in a respiratory sample (case) or not infected mother with no detection of the virus by PCR (control).

This analysis excluded severe symptomatic women who were referred to other hospitals, as previously mentioned.

For newborns, three groups were created according to the SARS-CoV-2 PCR result of the mother-neonate dyad from oropharyngeal swabs: 1) positive neonate of positive mother, 2) negative neonate of positive mother, and 3) negative neonate of negative mother.

### Primary and secondary outcomes

This study´s primary outcome was to describe the maternal/neonatal features and complications, including SARS-CoV-2 prevalence, in pregnant women with or without COVID-19 (asymptomatic or with mild/moderate symptoms). The secondary outcomes were associations with SARS-CoV-2 infection in these women.

### Statistical analysis

Descriptive statistics were reported as Median (Interquartile Range, IQR) for continuous variables or frequencies (percentage with 95% Confidence Intervals, CI) for categorical variables. All numerical variables were assessed for normality and outliers, and categorical variables were evaluated for multicollinearity. Mann-Whitney U test was used to compare continuous variables that were not normally distributed. The frequencies of categorical variables were analyzed using Pearson χ2 or Fisher’s exact tests. Binomial logistic regression analysis was performed to assess the crude/adjusted odds ratio and 95% CI of independent maternal risk factors for SARS-CoV-2 infection or maternal outcomes based on clinical variables. Odds ratios were adjusted for maternal Body Mass Index (BMI), age, comorbidities (such as hypertension, diabetes, autoimmune disease), and gestational age at the outcome as confounders, assessed by multivariable regression analysis. Neonatal data was only analyzed using Fisher’s exact tests. We did not carry out a sample size calculation since the primary objective was to describe SARS-CoV-2 positive patient´s features within our health care center in this exploratory study. Missing data were excluded in order to minimize inflating estimates. The data were analyzed by SPSS version 26.0 (SPSS Inc., Chicago, IL, USA). A value of *P*<0.05 was considered significant (2-sided).

## Results

### Maternal features

Over a 1-month period, a total of 250 pregnant women were attended for obstetric reasons without or with mild/moderate symptoms consistent with COVID-19. Among these, 70 pregnant women had a positive SARS-CoV-2 result, 170 women were negative for the test, and ten women were not tested, or the test result was not available, the latter were excluded from the study. Thus, the estimated prevalence of COVID-19 in this large tertiary level center in Mexico City was 29% (70/240, 95% CI, 24%-35%), similar to the 32.7% positivity rate in Mexico City during the study period. COVID-19 positive and negative pregnant women admittance were scheduled admission (43% vs. 45%) or labor (obstetric emergency, 57% vs. 52%), with no differences between both groups (*P* = 0.315).

The majority of SARS-CoV-2 positive women were asymptomatic (86%, 95% CI, 76%-92%), as described in other studies. Only ten positive women of 70 (14%, 95% CI, 8%-24%) presented mild to moderate symptoms consistent with COVID-19 (according to [13]), such as cough (3/70), fever (4/70), headache (4/70), myalgias (1/70), diarrhea (1/70), or dyspnea (2/70). None presented anosmia or ageusia. Two women in ten also presented cardiac arrhythmias, and one of them had mild pneumonia. Five pregnant women of 170 with a negative PCR result reported fever and 1 patient headache at admission.

Demographics and clinical features are presented in Table 1. The median age of COVID-19 infected and non-infected women were 26 and 29 years (IQR, 22–31 and IQR, 23–34, respectively) (*P* = 0.7). Only 16% of SARS-CoV-2 positive women and 20% of non-infected patients were aged >35. The median Body Mass Index of SARS-CoV-2 positive and negative women was 28.6 kg/m^2^ and 29.7 kg/m^2^ (IQR, 26.6–32.5 and IQR, 26.6–33.2, respectively) (*P* = 0.417); with more than 40% of women reported to have obesity. These numbers are similar to the obesity/overweight proportion observed in pregnant women at the national level.

**Table 1:** Maternal features of pregnant women with positive or negative SARS-CoV-2 RT-qPCR test.

Feature	Positive women (N = 70)	Negative women (N = 170)	OR (95% CI)	aOR (95% CI)**
	No. (%)*	No. (%)*		
Age, y				
<19	13 (19)	27 (16)	Referent group	Referent group
20–34	46 (66)	109 (64)	0.9 (0.4–1.8)	1.2 (0.5–2.7)
>35	11 (16)	34 (20)	0.7 (0.2–1.7)	1.1 (0.4–2.9)
Median, range	26, 13–45	29, 13–45		
Missing data	0	0		
Occupation				
At home	41 (59)	100 (58)	Omitted	Omitted
Employee	3 (4)	10 (6)		
Student	3 (4)	2 (1)		
Pre-graduate	2 (3)	1 (<1)		
Unknown	21 (30)	57 (33)		
Missing data	0	0		
Body Mass Index, kg/m^2^				
Normal	13 (20)	25 (16)	Referent group	Referent group
Overweight	27 (41)	56 (35)	0.9 (0.4–2)	1 (0.4–2.3)
Obese	26 (39)	78 (49)	0.6 (0.3–1.4)	0.7 (0.3–1.6)
Median, range	29, 19–57	30, 19–55		
Missing data	4	11		
Pre-existing comorbidities				
None	59 (84)	128 (75)	Referent group	Referent group
Any	11 (15)	42 (23)	0.6 (0.3–1.2)	(0.5 (0.2–1.2)
Hypertension	2 (3)	8 (5)	0.5 (0.1–2.6)	0.4 (0.1–2.3)
Asthma	0	2 (1)	0	0
Cardiac disease	1 (1)	2 (1)	1 (0.1–12)	1 (0.1–11.7)
Arthritis	2 (3)	2 (1)	3.2 (0.5–20)	1.8 (0.2–13)
Diabetes mellitus	2 (3)	10 (6)	0.6 (0.2–2.4)	0.2 (0.02–1.6)
Hypothyroidism	3 (4)	18 (10)	0.1 (0.02–1.1)	0.3 (0.09–1.1)
Lupus	1 (1)	0	omitted	omitted
Missing data	0	0		
Multiple pregnancy	3 (4)	13 (8)	0.5 (0.1–1.9)	0.6 (0.1–2.5)
Missing data	0	0		
Gestational Diabetes	5 (7)	12 (7)	1 (0.3–3)	2.2 (0.6–8.6)
Missing data	0	0		
Gestational age at the time of admission, w				
<24	7 (10)	10 (6)	Referent group	Referent group
25–27	0	8 (5)	0	0
28–32	8 (11)	10 (6)	1.1 (0.3–4.3)	1.1 (0.2–5.2)
33–36	9 (13)	27 (16)	0.5 (0.1–1.6)	0.5 (0.1–2.3)
>37	46 (66)	113 (67)	0.6 (0.2–1.6)	0.5 (0.2–2.2)
Median, range	38, 16–41	38, 7–42		
Missing data	0	2		
Smoking	1 (<1)	3 (2)		Omitted
Missing data	5	10		
Gravida				
1	26 (37)	53 (31)	Referent group	Referent group
2	30 (43)^a^	46 (27)^b^	1.4 (0.7–2.7)	1.3 (0.6–2.9)
>3	14 (20)^a^	70 (41)^b^	0.4 (0.2–0.9)	0.5 (0.2–1.2)
Median, range	2, 1–5	2, 0–10		
Missing data	0	1		
Para				
0	56 (81)^a^	109 (64)^b^	Referent group	Referent group
1	11 (16)	43 (25)	0.5 (0.2–1.1)	0.6 (0.3–1.5)
>2	2 (3)	18 (11)	0.3 (0.1–1.2)	0.6 (0.1–2.5)
Median, range	0, 0–3	0, 0–9		
Missing data	0	1		
Abortion				
0	47 (67)	105 (61)	Referent group	Referent group
1	18 (26)	42 (25)	0.9 (0.5–1.8)	0.9 (0.4–2)
>2	5 (7)	24 (14)	0.4 (0.1–1.2)	0.6 (0.2–1.8)
Median, range	0, 0–9	0, 0–4		
Missing data	0	0		
Previous cesarean				
0	37 (54)	100 (59)	Referent group	Referent group
1	20 (29)	42 (25)	1.3 (0.7–2.5)	1.5 (0.7–3.1)
2	9 (13)	23 (13)	1 (0.4–2.4)	0.9 (0.3–2.5)
>3	3 (4)	5 (3)	1.6 (0.4–7.3)	0.9 (0.1–5.6)
Median, range	0, 0–3	0, 0–3		
Missing data	1	0		

*Percentages calculated excluding missing values

**Adjusted for maternal age, BMI, pre-existing comorbidities, gestational age at the time of admission. Each subscript letter denotes a subset of pregnant women category whose column proportions do not differ significantly from each other at the *P*<0.05 level in crosstab analysis.

The frequency of pre-existing chronic diseases was 16% and 25% for SARS-CoV-2 infected and non-infected patients (95% CI, 19% to 32% vs. 9% to 25%, *P* = 0.312). Diabetes mellitus, hypertension, arthritis, and hypothyroidism were the most commonly reported underlying chronic disorders, without differences between both groups of women.

Concerning conditions associated with pregnancy, a total of 7% in each group developed gestational diabetes (95% CI, 3%-15% in positive and 4%-12% in negative women, respectively) (*P* = 0.587). More than 60% of both groups of pregnant women had two or more previous pregnancies.

Maternal age >35 years, obese BMI, multiple pregnancy, and gestational diabetes were not found associated with SARS-CoV-2 positive women, as evaluated by logistic regression models (Table 1).

### Maternal and perinatal outcomes

Primary and secondary outcomes are depicted in Table 2. Both groups of women were admitted at the hospital for delivery mostly during the third trimester (89%), the median gestational age at the end of pregnancy was 38.1 weeks for both groups of women (IQR, 36.3–39.3 and IQR, 36.3–39.1 weeks, for SARS-CoV- 2 positive and negative women, respectively, *P* = 0.769). The mode of delivery was C-section in more than 70% of both groups; of these, 33% positive vs. 26% negative women were due to maternal indications, 16% vs. 19% obstetric, and 25% vs. 20% to fetal signs (*P* = 0.538). The baseline C-section rate for Mexico City is 35% and at this particular healthcare facility is 54%. However, we observed an increase in the rate of C-sections (70%) during the COVID-19 pandemic compared to other years. At the time of this analysis, 219 pregnancies were completed (95.3%); 76% of deliveries occurring at term. Premature birth did not differ between women infected or not with COVID-19 (22%, 95% CI, 13%-33% vs. 24%, 95% CI, 18%-31%) (*P* = 0.861).

**Table 2:** Maternal and pregnancy outcomes.

Outcomes	Positive women (N = 70)	Negative women (N = 170)	OR (95% CI)	aOR 95% CI**
	No. (%)*	No. (%)*		
Critical Care	1 (<1)	0	Omitted	Omitted
Missing data	0	0		
Pneumonia	1 (<1)	0	Omitted	Omitted
Missing data	0	0		
Ongoing pregnancy	2 (3)	9 (5)	Omitted	Omitted
Pregnancy completed	62 (97)	157 (94)		
Missing data	6	4		
Hospital stay, d				
1–2	57 (84)	148 (88)	Referent group	Referent group
3–5	8 (12)	16 (9)	1.3 (0.5–3.2)	1.4 (0.5–4.5)
>6	3 (4)	4 (2)	1.9 (0.4–9)	1.5 (0.2–10.8)
Median, range	1, 0–7	2, 0–11		
Missing data	2	2		
Preeclampsia	12 (18)^a^	15 (9)^b^	2.2 (1.003–5.2)	2.1 (0.8–5.2)
Missing data	3	6		
Hemorrhage	4 (6)	6 (3)	Omitted	Omitted
Missing data	1	1		
Premature rupture of membranes	8 (12)	18 (11)	1.1 (0.4–2.6)	1.1 (0.5–3.1)
Missing data	4	5		
Gestational age at delivery, w				
<24	3 (4)	6 (4)	Referent group	Referent group
25–27	2 (3)	4 (2)	0.5 (0.04–6.7)	0.9 (0.05–14)
28–32	7 (11)	7 (4)	2 (0.3–11)	1.6 (0.2–11)
33–36	6 (9)	28 (18)	0.4 (0.08–2.2)	0.4 (0.06–2. 5)
>37	48 (73)	111 (71)	0.9 (0.2–3.6)	1.1 (0.2–5.1)
Median, range	38.1, 19–41.5	38.1, 20.2–42.1		
Missing data	4	14		
Preterm birth	14 (22)	38 (24)	0.9 (0.4–1.7)	1.2 (0.2–6.6)
Term birth	50 (78)	119 (76)	Referent group	Referent group
Missing data	6	13		
Mode of Delivery				
Cesarean	51 (81)	113 (73)	Referent group	Referent group
Labor	12 (19)	41 (27)	0.6 (0.3–1.3)	0.6 (0.2–1.7)
Missing data	7	16		
Indications for delivery				
Maternal^1^	26 (40)	54 (35)	Referent group	Referent group
Obstetric^2^	20 (31)	59 (39)	0.7 (0.3–1.4)	0.7 (0.3–1.5)
Fetal^3^	18 (28)	39 (26)	0.9 (0.5–1.9)	0.9 (0.4–2.1)
Missing data	6	18		
Miscarriage	3 (4)	3 (2)	Omitted	Omitted
Antepartum stillbirth	1 (1)	1 (<1)		
Intrapartum stillbirth	1 (1)	2 (1)		
Total fetal death	5 (7)	6 (3)		
Missing data	0	0		
Live birth	63 (92)	155 (96)	Omitted	Omitted
Missing data	2	9		
Final Outcome			Omitted	Omitted
Discharged well	70 (100)	170 (100)		
Died	0	0		

*Percentages calculated excluding missing values

**Adjusted for maternal age, BMI, pre-existing comorbidities, gestational age at the time of admission.

^1^preeclampsia, supraventricular maternal tachycardia, intrahepatic cholestasis of pregnancy, systemic lupus erythematosus

^2^fetal distress or abnormal presentation, congenital defect, intrauterine growth restriction, multiple gestation, and

^3^previous cesarean, premature rupture of membranes, abruptio placentae, fetal pelvic presentation. Each subscript letter denotes a subset of pregnant women category whose column proportions do not differ significantly from each other at the *P*<0.05 level in crosstab analysis.

Three of 70 SARS-CoV-2 positive women were admitted at the care center for more than six days (4%, 95% CI, 1%-11%) for uncontrolled hypertension, lupus, or prematurity in comparison to four of 170 negative women (2%, 95% CI, 1%-6%) (*P* = 0.769). Only one COVID-19 patient with mild pneumonia and cardiac arrhythmia was admitted to the intensive care unit; she developed severe preeclampsia and delivered a live newborn at term. As a global outcome, all women were discharged at home without complications, and no maternal deaths were reported.

Regarding other adverse maternal outcomes, premature rupture of membranes was reported in 12% COVID-19 positive women (95% CI, 6%-22%) compared to 11% negative patients (95% CI, 7%-17%, *P* = 0.921). A significantly increased number of SARS-CoV-2 women presented preeclampsia (12/70, [18%], 95% CI, 10%-29%) in contrast with negative women (15/170, [9%], 95% CI, 5%-14%, *P*<0.05). The logistic regression model found that infected women were 2.2 times more likely to develop this complication (OR = 2.2, 95% CI, 1.003–5, *P* = 0.049). This association did not persist in the sensitivity analysis when adjusted for maternal age, BMI, and pre-existing diseases (OR 2.1, 95%CI, 0.8–5.2) or for maternal age, BMI, and hypertension (OR 2, 95% CI 0.9–4.8).

Concerning fetal loss, 5 cases of 70 (8%, 95% CI, 3%-16%) from infected mothers and 6 of 170 negative mothers (4%, 95% CI, 2%-8%) were reported; three miscarriages and one antepartum stillbirth in each group of women; one intrapartum stillbirth in an infected woman and two in negative women; with no statistical differences in the total of fetal deaths (*P* = 0.305). The causes of fetal deaths were as follows: in the group from infected mothers, one had an antepartum fetal death at 36.6 weeks of pregnancy, we were not able to establish a definitive cause, the placenta was reported as hypotrophic with decidual vasculopathy; the other patient had an intrapartum fetal death secondary to premature rupture of membranes at 24.4 weeks of pregnancy. In the negative group, one patient presented an antepartum fetal death at 23 weeks; the fetus had a prenatal diagnosis of cardiopathy, intrauterine growth restriction, and anhydramnios. Two patients had intrapartum fetal death, one case presented at 23.1 weeks of pregnancy with premature rupture of membranes and chorioamnionitis, and the mother of the second case presented at 26.3 weeks with premature labor; the placenta showed mild chorioamnionitis.

### Neonatal outcomes

From 217 neonates born alive, we report the results of 39 infants that had a SARS-CoV-2 test performed within 24 hours of birth, according to the classification system of SARS-CoV-2 infection [16]. Newborns with a PCR test > 24h or without a result were therefore excluded from this study. We analyzed the outcomes for neonates of SARS-CoV-2-confirmed mothers: nine positive neonates (23%) and twenty-one negative neonates (54%), as well as nine negative neonates from negative mothers who presented symptoms such as fever or headache (23%) (Table 3).

**Table 3:** Neonatal characteristics and outcomes.

Feature	Positive mother Positive neonate (N = 9)	Positive mother Negative neonate (N = 21)	Negative Mother Negative neonate (N = 9)	*P*
	No. (%)			
Gestational age at birth				0.496
<34	1 (11)	1 (5)	2 (22)	
34–36	3 (33)	4 (19)	2 (22)	
>37	5 (56)	16 (76)	5 (56)	
Median, range, w	38.2, 28–39.5	38.2, 29–41.6	37.1, 32.2–40.4	
Missing data	0	0	0	
Birth weight				0.340
<1500	1 (11)	1 (5)	2 (22)	
1500–2499	3 (33)	3 (14)	2 (22)	
>2500	5 (56)	17 (81)	5 (56)	
Median, range, g	2850, 1050–4038	3170, 1050–3905	2696, 1275–3720	
Missing data	0	0	0	
Birth weight for gestational age				0.042*
Appropriate-for-gestational-age	3 (33)^a^	17 (81)^b^	7 (78)^a,b^	
Small-for-gestational-age	4 (44)	4 (19)	2 (22)	
Large-for-gestational-age	2 (22)	0	0	
Missing data	0	0	0	
Sex				0.682
Female	4 (50)	11 (52)	3 (8)	
Male	4 (49)	10 (48)	6 (15)	
Undetermined	1	0	0	
Missing data	0	0	0	
Apgar at minute 1				0.896
1–3	0	1 (5)	0	
4–6	2 (22)	2 (9)	1 (12)	
7–9	7 (78)	18 (86)	7 (87)	
Missing data	0	0	1	
Apgar at minute 5:				0.660
1–3	0	1 (5)	0	
7–9	9 (100)	20 (95)	8 (100)	
Missing data	0	0	1	
Antenatal glucocorticoid	3 (33)	2 (9)	2 (22)	0.303
Missing data	0	0	0	
Reanimation:				0.168
None	4 (44)	9 (43)	1 (11)	
Oxygen	1 (11)	8 (38)	5 (56)	
Oxygen+CPAP	2 (22)	1 (5)	2 (22)	
Oxygen+Face mask ventilation+CPAP	1 (11)	3 (14)	1 (11)	
Oxygen+Face mask ventilation+ETT ventilation	1 (11)	0	0	
Missing data	0	0	0	
Surfactant	1 (11)	0	0	Omitted
Missing data	0	0	0	
Admitted to NICU	4 (44)^a^	1 (5)^b^	2 (22)^a,b^	0.031*
Missing data	0	0	0	
Ventilation:				0.224
None	6 (15)	15 (71)	4 (44)	
Oxygen	0	4 (19)	3 (33)	
Conventional mechanical ventilation	1 (11)	0	0	
Continuous Positive Airway Pressure	2 (22)	2 (9)	2 (22)	
High Frequency Ventilation	0	0	0	
Missing data	0	0	0	
Respiratory morbidities:				0.024*
None	6 (67)^a,b^	20 (95)^a^	5 (56)^b^	
Transitory Tachypnea	0	0	1 (11)	
Respiratory Distress Syndrome	2 (22)	1 (5)	2 (22)	
Tachypnea	0	0	1 (11)	
Pulmonary Hypertension	1 (11)	0	0	
Missing data	0	0	0	
Gastrointestinal morbidities:				Omitted
None	8 (88)	21 (100)	9 (100)	
NEC	1 (1)	0	0	
Missing data	0	0	0	
Malformations:				Omitted
CNS	0	1 (5)	0	
Multiple (CNS, urogenital, cardiac)	1 (11)	1 (5)	0	
Missing data	0	0	0	
Days at hospital:				<0.001*
1–2	5 (56)^a,b^	18 (90)^a^	4 (44)^b^	
3–6	0	2 (10)	3 (33)	
>7	4 (44)^a^	0^b^	0^a,b^	
Median, range, d	2, 1–23	2, 0–3	2, 1–4	
Missing data	0	1	2	
Final Outcome:				0.702
Discharged at home	8 (89)	18 (90)	5 (55)	
Discharged to hospital	0	1 (5)	0	
Still admitted	0	1	2 (6)	
Died	1 (11)	1 (5)	2 (6)	
Missing data	0	0	0	
Feeding at discharge:				0.066
Fasting	0	1 (5)	0	
Human milk	5 (62)	12 (63)	5 (71)	
Human milk + Formula	3 (37)^a^	0^b^	1 (14)^a,b^	
Formula	0	6 (32)	1 (14)	
Missing data	1	2	2	

Each subscript letter denotes a subset of pregnant women category whose column proportions do not differ significantly from each other at the *P*<0.05 level in crosstab analysis.

* *P*<0.05.

The median gestational age (GA) of positive and negative neonates of SARS-CoV-2-positive mothers was 38.2 weeks (IQR, 35–38.4 weeks and 37.4–39.5 weeks, respectively), and 37.1 weeks for neonates of negative mothers (IQR, 32.6–39.8 weeks) (*P* = 0.486). Forty-four percent of COVID-19 positive neonates (95% CI, 11%-65%) were preterm compared to 24% negative newborns of infected women (95% CI, 7%-59%) (*P* = 0.496). The median birth weight of positive neonates was 2850 g (IQR, 1770-3470g), 3170 g (IQR, 2505-3320g) for negative neonates of SARS-CoV-2-mothers, and 2696 g (IQR, 1847-3345g) for infants of negative mothers (*P* = 0.340). However, when classifying weight by gestational age, a significantly increased number of large- and small-for-gestational-age (LGA or SGA) infants were found in the group of positive neonates in contrast to negative neonates of SARS-CoV-2-mothers (2/9 LGA, [22%] vs. 0/21 LGA [0%] and 4/9 SGA, [44%] vs. 4/21 SGA [19%]), and newborns from negative mothers (0/9, [0%] and 2/9 [22%]) (*P*<0.05). Nevertheless, when comparing LGA or SGA infants separately between the three groups of neonates, only LGA infants were found significantly increased in positive infants compared to the other two groups of neonates (*P* = 0.03 and *P* = 0.332 for LGA and SGA, respectively). However, the number of neonates was very small.

Most neonates were only admitted for 1–2 days (71%). COVID-19 positive neonates had longer hospital stay (mean, [IQR], 2 [2–18] days) compared to uninfected neonates of positive or negative mothers (2 [1–2] days and 2 [2–3] days, *P*<0.001), with four positive neonates of 9 hospitalized for more than 7 days compared to none in the other two groups (44%, 95% CI, 17%-75%, *P*<0.05). Of these, the reasons for admission were pulmonary hypertension, acute respiratory distress syndrome (ARDS), pneumonia, or sepsis. In the COVID area, a higher number of SARS-CoV-2 positive infants were admitted to the NICU in comparison with negative newborns from infected or uninfected mothers (4/9, [44%], 95% CI, 17%-75% vs. 1/21, [5%], 95% CI, 0.5%-20%, vs. 2/9, [22%], 95% CI, 5%-54%, respectively, *P*<0.05).

In terms of malformations, no cases were reported for neonates from uninfected mothers compared to one COVID-19 positive neonate of 9 and two negative neonates of 21 from infected mothers (multiple malformations or a congenital CNS malformation (encephalocele)).

More than fifty percent of positive and negative neonates from infected or uninfected women did not require respiratory support or only oxygen at birth (5/9, [55%] vs. 17/21, [81%] vs. 6/9, [66%], *P* = 0.168) or during hospitalization (6/9, [66%] vs. 19/21 [90%] vs. 7/9, [78%], *P* = 0.222). The majority of neonates did not present any respiratory disease; 67% positive newborns (95% CI, 35%-89%), 95% negative neonates (95% CI, 79%-99%) of infected mothers or 55% negative neonates of negative mothers (95%CI, 25%-83%, *P*<0.05). Respiratory distress syndrome was reported in two neonates with COVID-19 infection (<34 weeks and 34–36 weeks GA), one negative neonate of an infected woman (<34 weeks GA), and two newborns of negative mothers (34–36 weeks GA). Only one SARS-CoV-2 positive neonate presented pulmonary hypertension (34–36 weeks). Overall, respiratory morbidities were more common in positive neonates, with a statistically significant difference between the three groups (*P* = 0.024).

As an outcome, most neonates were discharged at home without complications (85%), one and two COVID-19 negative neonates from positive and negative mothers, respectively, were still admitted at the time of this analysis (they were born May 23, 2020). Four deaths were observed during the neonatal period, 2 cases in infected mothers and 2 in uninfected women. One positive neonate and one negative neonate of COVID-19-mothers died (septic shock and multiple congenital disabilities, respectively). One infant died at 24.3 weeks of gestation in the negative group because of prematurity and the other newborn at 40 weeks secondary to neonatal asphyxia.

## Discussion

### Maternal characteristics and outcomes

After universal screening over a period of one month of deliveries in this center attending obstetric admissions without severe COVID-19 symptoms, we report a prevalence of 29% of SARS-CoV-2 percent positivity, of these, 86% were asymptomatic. Most pregnant women were admitted during the third trimester; the demographics and clinical characteristics of COVID-19 infected women were similar to uninfected women, including maternal age, Body Mass Index, and pre-existing diseases. No differences in perinatal outcomes were found between SARS-CoV-2 positive and negative pregnant women, except for preeclampsia. All women were discharged at home without complications, and no maternal death was reported.

Our estimated prevalence of SARS-CoV-2 infection in pregnant women is higher than rates reported worldwide (13%-20%) after universal screening [11,12,17–19]. Indeed, at the time of this study, the positivity rate in Mexico City was 32.7% and 47% at a national level, which is in accordance with the reported high prevalence of pregnant women in this center.

One possible explanation for the increased COVID-19 prevalence among pregnant patients is that some of the clinical manifestations of SARS-CoV-2 infection in pregnant women overlap with symptoms of normal pregnancy (such as fatigue, PRIORITY Study) [20] or complications of obstetric disorders (headache, fever, findings in preeclampsia, gastrointestinal illness) [21].

More than 85% of pregnant women admitted for childbirth that tested positive for SARS-CoV-2 were asymptomatic. This is in accordance with studies from universal screening in distinct New York City hospitals [22,23] and other U.S. Health care centers [9], but is different from the results presented by two delivery units in Los Angeles that found 13 to 14% [11,24]), 4% in a tertiary center in Tokyo [19], or 32% in a tertiary care center in New York [25]. Our reported rates of asymptomatic women should be interpreted with caution since this center does not admit for obstetric reasons pregnant women with severe symptoms (as a non-COVID-19 hospital), and the overestimation of non-symptomatic cases is likely (symptomatic pregnant women were referred to COVID-19 hospitals).

Several studies, including a large national cohort study in the UK (UKOSS) and in the U.S. (CDC), reported maternal age, ethnicity, or obesity to be associated with COVID-19 severity compared with control pregnant women from retrospective cohorts [5], non-pregnant women [26,27], PCR negative women [24], symptomatic/asymptomatic pregnant women [8,9] and a systematic review and meta-analysis [10]. This referral center only attends complicated pregnancies, and as such, both groups of women (infected vs. non-infected patients) have risk factors associated with the gravity of COVID-19 illness (i.e., maternal age >35 years, obesity, and pre-existing disorders) compared to pregnant women in other health care centers.

We did not observe the association of SARS-CoV-2 infection with complications such as hemorrhage, premature rupture of membranes, or preterm birth. One possibility is that infected women were mostly asymptomatic, which is probably related to improved pregnancy outcomes in this cohort. Several studies have reported limited adverse outcomes in pregnant women [2–6]. However, other authors have noted in severe cases of COVID-19 infection longer days of hospitalization [28] and maternal morbidity, including death [8,9,29–31]. Is it also conceivable that part of the population attended at this center, which is a non-COVID-19 facility, were obstetric emergencies or scheduled admissions that normally would have been admitted in other hospitals but because patients are not symptomatic, they were referred to this center, increasing the adverse outcomes of the control group. Another possible explanation is that no differences were found when comparing outcomes in PCR positive vs. negative patients since both groups have similar underlying health problems. Also, pregnant women with underlying diseases (such as diabetes, hypertension, arthritis, and hypothyroidism) had an adequate management that led to proper control of these disorders improving the outcomes of pregnancies in both groups.

An increased number of SARS-CoV-2 women developed preeclampsia. In this sense, a study is being conducted in our center at the placental, clinical, biochemical, and ultrasound levels to characterize this observation further. A preeclampsia-like syndrome has recently been found in severe COVID-19 pregnancies based on differences in angiogenic factors such as the ratio of the soluble fms-like tyrosine kinase-1 and placental growth factor (sFlt-1/PlGF) [32]. Preeclampsia diagnosis in our center is performed under the international clinical and laboratory criteria [33], however, the measurement of sFlt-1/PlGF is not a routine index for its diagnosis. It may be questioned if patients diagnosed with SARS-CoV-2 and preeclampsia presented the syndrome as a manifestation of COVID-19 or placental damage increased by the viral infection that triggered the symptoms. In subsequent studies, it may be asked whether the sFlt-1/PlGF index could be used as a differential diagnosis.

Perinatal death has been observed in 7% of mothers infected by SARS-CoV-2, including one stillbirth in a mother with pneumonia [34] and one neonatal death in a baby who developed refractory shock and gastric bleeding with disseminated intravascular coagulation [35]. We observed four perinatal deaths in the COVID-19 positive group, one stillbirth secondary to premature rupture of membranes, reported in 26% of infected mothers [35,36], and two neonatal deaths of which one baby was born prematurely, developed pneumonia, and septic shock. He was positive for SARS-CoV-2 within 24h of birth. We did not find differences between our groups; however, we need to study more patients to confirm this finding.

The relevance of the high positivity index in asymptomatic women is two-sided: 1) In hospitals were universal screening is not performed, it could be recommended that contention measures should include managing every patient with unknown infectious status as potentially infected for the protection of the neonate and the medical staff involved in their attention in areas of high transmission; 2) The current lack of knowledge of the virus behavior and its long-term effects demand a strict follow-up of the infants of asymptomatic women from the neurological development point of view since this virus is also neurotropic [37]. If it is not feasible to determine viral infection in pregnant women, the possibility of studying the short and long effects, including the neurocognitive features of their children, will be lost.

### Neonatal outcomes

In this study, of the 70 SARS-CoV-2 positive mothers, 63 had a live newborn, and of these, only 30 babies had an oropharyngeal swab in the first 24h of life, of which 9 had a positive test. The approximate prevalence of newborns with a positive test was 12.8%, much higher than that reported in other countries where most newborns had a negative test [5,38–41] such as in Spain, where they reported 3 of 42 SARS-CoV-2 positive infants, who negativized in the first 24 hours and took them as false-positive results [38–40]. However, a recent study from the CDC indicated a 2.6% percent positivity among newborns with a molecular test reported [7]. It must be pointed out that neonates were not universally screened; most of these cases were newborns from positive mothers and a small number of neonates of negative mothers who had symptoms such as fever or headache. In consequence, the high percent positivity of infants is probably overestimated. It is important to mention that this study´s intent was not to determine whether or not neonates can vertically acquire SARS-CoV-2 through intrauterine maternal transmission but rather to characterize neonatal outcomes.

At birth, the gestational age was similar to that mentioned by other centers, the vast majority being term infants [5,38–40]. Unlike other studies [30,41], no difference was found in the number of preterm births between COVID-19 positive and negative mothers and in neonatal resuscitation requirements during birth between the groups. This could be explained by the limitation/difference of this study of analyzing only those hospitalized and with mild/asymptomatic infections.

It is important to emphasize that we found a significant difference in the number of newborns admitted to the NICU, which was greater in the SARS-CoV-2 positive group, given by a higher number of infants with respiratory disease in this group and in longer hospital stay: these are distinct proxies for illness severity. This contrast with the data reported in the UKOSS study, where only one newborn with a positive test before 24 hours of life was hospitalized in the NICU [5]. However, it is not straightforward to compare our neonatal outcomes with other studies since very few centers have reported positive newborns tested within 24h of life [5,38–40]. However, a recent systematic review found a relatively higher number of neonates born to COVID-19 mothers admitted to the NICU [10].

It is important to clarify that of the four SARS-CoV-2 positive neonates that required longer hospitalization days, one was a term neonate that presented several congenital anomalies from a mother with hypothyroidism, the other three newborns were twins of 35 weeks GA that presented ARDS or pulmonary hypertension, and one neonate of 28 weeks GA with pneumonia, early sepsis who died from septic shock. Negative neonates admitted to the NICU, were premature; of these, one infant was 29 weeks GA, and the others were twins of 32 weeks GA that presented ARDS. Thus, the difference in hospital length-of-stay or admission to NICU between COVID-19 positive and negative neonates did not seem to be due to an increased number of newborns with malformations or prematurity in the positive group. However, the number of neonates is small, which requires further investigation. Whether the neonatal morbidity observed in positive neonates is associated with SARS-CoV-2 infection and increased compared to uninfected infants is unknown. Nevertheless, the results presented in this study suggest there are complications in premature infants with COVID-19 infection and that careful assessment of infected neonates is justified.

### Limitations of the study

This study has several limitations. First, it is a single-center study with a modest sample size for pregnant women. As well, we have to acknowledge the small number of SARS-CoV-2 tested neonates, the majority of positive mothers and a reduced number of infants of negative mothers with symptoms, such as fever or headache. Neonates were not universally tested in this center. Therefore, the neonatal percent positivity is likely an overestimate if the results are not reported for the entire population. Second, only mother-neonate dyads with a SARS-CoV-2 PCR result were included in the study resulting in a recruitment bias. Third, this center did not admit for delivery pregnant women with severe COVID-19 symptoms (non-COVID-19 hospital); therefore a bias existed in the proportion of asymptomatic patients. Fourth, pregnant women usually attended in other hospitals for obstetric reasons but that did not show severe symptoms were referred to this health care center, thus probably increasing the percent positivity. Fifth, the rate of COVID-19 diagnosis varies between studies depending on the sampling strategy, which was universal screening in this center compared to sampling based on symptoms. Sixth, true asymptomatic and pre-symptomatic women were not differentiated in this analysis since this is another study’s objective. Seventh, high-risk obstetric admissions in this center limit comparison of basal characteristics and outcomes with all pregnant women i.e., normal pregnancies. Eight, the high prevalence of COVID-19 infection in pregnant women found in this study may not be comparable to other countries such as the U.S. (8%) or Argentina (9.4%) partially because the SARS-CoV-2 local infection rate is different. Also, it is possible that these values are increased because testing in Mexico was mainly targeted at people with severe COVID-19 symptoms and a substantial underdiagnosis (fewer tests for every confirmed case) compared to other countries. Additionally, this care center also attended patients referred from COVID-19 hospitals that did not present severe illness and were therefore admitted for obstetric reasons in this center, thereby increasing the percent positivity. Finally, there is also the possibility of false negatives reported [42] in pregnancy [43] and which can underestimate the outcomes.

We are cautious of generalizing the results; nonetheless the close assessment of the perinatal outcomes is warranted.

### Conclusions

Although the prevalence of COVID-19 was high among pregnant women in this health care center, it is reassuring that no differences in most adverse outcomes were found among women with asymptomatic and mild SARS-CoV-2 infection, even among patients with underlying comorbidities associated with severe disease. However, this study found an association of increased preeclampsia with SARS-CoV-2 infection that needs to be further evaluated.
